# Enhancing bridge damage detection with Mamba-Enhanced HRNet for semantic segmentation

**DOI:** 10.1371/journal.pone.0312136

**Published:** 2024-10-16

**Authors:** Jie Liu, Deyuan Li, Xin Xu

**Affiliations:** 1 Sichuan University Jinjiang College, Meishan, China; 2 School of Statistics and Mathematics, Yunnan University of Finance and Economics, Kunming, China; Bayer Crop Science United States: Bayer CropScience LP, UNITED STATES OF AMERICA

## Abstract

With the acceleration of urbanization, bridges, as crucial infrastructure, their structural health and stability are paramount to public safety. This paper proposes Mamba-Enhanced HRNet for bridge damage detection. Mamba-Enhanced HRNet integrates the advantages of HRNet’s multi-resolution parallel design and VMamba’s visual state space model. By replacing the residual convolutional blocks in HRNet with a combination of VSS blocks and convolution, this model enhances the network’s capability to capture global contextual information while maintaining computational efficiency. This work builds an extensive dataset with multiple damage kinds and uses Mean Intersection over Union (Mean IoU) as the assessment metric to assess the performance of Mamba-Enhanced HRNet. Experimental results demonstrate that Mamba-Enhanced HRNet achieves significant performance improvements in bridge damage semantic segmentation tasks, with Mean IoU scores of 0.963, outperforming several other semantic segmentation models.

## 1 Introduction

With the advancement of urbanization, the health and stability of bridges, as crucial components of infrastructure, have significant implications for public safety and property. However, due to natural and human factors, bridge structures may experience various forms of damage, such as cracks and corrosion. If these damages are not detected and addressed promptly, they can lead to a decline in structural performance or even catastrophic bridge failures. Therefore, regular crack detection and proactive measures to prevent potential safety incidents are critical issues in the field of civil engineering.

Traditionally, crack detection in bridges has relied primarily on manual inspections. However, this method is inefficient and highly dependent on the experience and subjective judgment of the inspectors, rendering it inadequate for large-scale and high-efficiency detection needs. Consequently, the application of modern information technology to achieve automated and intelligent detection of bridge damage has become a pressing issue.

Artificial intelligence technology has advanced quickly in recent years and produced notable outcomes in a number of domains, including speech and image recognition. Artificial intelligence (AI) has become a popular tool in image recognition, outperforming conventional methods in medical imaging analysis, object detection, and facial recognition. A crucial deep learning technique called semantic segmentation seeks to provide a category label to every pixel in an image so that the content of the image may be fully understood. In the context of bridge damage, semantic segmentation can accurately distinguish between damaged and non-damaged areas, providing robust support for damage identification and measurement.

Currently, several mainstream semantic segmentation algorithms, such as U-Net [1], DeepLabV3+ [2], and HRNet [3], have been widely applied in this field due to their excellent performance. Specifically, HRNet, through multi-scale feature fusion, maintains a high-resolution representation of images, providing rich contextual information for crack segmentation and enhancing the network’s ability to detect small cracks.

Mamba, an advanced state space model, is designed to capture long-term dependencies in time series. Based on this, VMamba [4], with its innovative cross-scan module, not only retains the advantages of global receptive fields and dynamic weights but also achieves linear complexity. This makes it particularly effective in processing high-resolution images and demonstrates its outstanding performance in image recognition and analysis.

Based on the aforementioned research background and technological advancements, this paper explores the application of Mamba technology in bridge damage detection. It attempts to integrate Mamba with the HRNet semantic segmentation algorithm to achieve higher crack detection accuracy, thereby introducing innovative technical means to bridge maintenance in the field of civil engineering.

The primary contributions of this paper are as follows:

1. A novel deep learning model that integrates the advantages of VMamba and HRNet is proposed, enhancing the accuracy of bridge damage detection.
2. Extensive tests were conducted using publicly available datasets to validate the superiority and effectiveness of the proposed approach.

The remainder of the paper is organized as follows: Section 2 reviews the literature on bridge crack and damage detection. Section 3 details the proposed approach. The experimental results are presented in Section 4. Finally, Section 5 concludes with a discussion of the findings.

## 2 Related work

### 2.1 CNN-based methods

Convolutional neural networks (CNNs) have demonstrated robust performance in image processing and computer vision, particularly in image semantic segmentation tasks. In recent years, researchers have significantly enhanced the ability of CNNs to capture detailed features by introducing innovative structures and modules. Fu et al. [5] introduced an improved DeepLabv3+ semantic segmentation algorithm for bridge crack detection. By incorporating a densely connected atrous spatial pyramid pooling module, the method achieves denser pixel sampling, significantly boosting the network’s capability to capture detailed crack features. The BC-DUnet, a bridge crack segmentation network proposed by Liu et al. [6], efficiently enhances the saliency of small cracks by attenuating background features. Key characteristics of minor cracks are highlighted while irrelevant data is filtered out through the integration of a background elimination module, a cross-attention mechanism, and a dense linked feature extraction model within the BC-DUnet framework. In order to detect cracks and approximate their locations in multi-object photographs, Jian Zhang et al. [7] curated a dataset and refined the YOLO method, resulting in the creation of CR-YOLO. Furthermore, they enhanced the PSPNet algorithm to distinguish areas devoid of bridge cracks from those exhibiting such cracks. Addressing the challenge of data annotation and improving model generalization for crack identification and assessment, Zheng et al. [8] proposed a multistage semi-supervised active learning framework known as CAL-ISM. To enhance the detection performance of fine cracks, a bridge crack segmentation approach was introduced by Yuan et al. [9], leveraging a parallel attention mechanism and multi-scale feature fusion. Considerable reduction in the parameter count of the DeepLabv3+ network was achieved, accompanied by heightened training and prediction speed, achieved through the integration of an enhanced lightweight MobileNetv2 network with dilated separable convolution. Sun et al. [10] outlined an integration-competition network (CCSNet) designed for bridge crack segmentation within complex scenarios. This network addresses challenges such as high-frequency light, intricate backgrounds, and microscopic fissures, all of which can compromise segmentation accuracy.

### 2.2 Transformer-based methods

With the tremendous success of Transformer models in natural language processing (NLP), their application in computer vision tasks has garnered increasing attention. Transformer models address long-range dependency issues through self-attention mechanisms, providing new perspectives for image recognition and segmentation tasks. The Vision Transformer, introduced by Dosovitskiy et al. [11], was developed specifically for image recognition tasks. ViT has demonstrated comparable performance to CNN-based approaches through pre-training on extensive datasets and utilizing 2D image patches along with positional embeddings as input. Liu et al. [12] initially introduced the Swin Transformer, a hierarchical model that employs shifted windows to effectively compute feature representations. When utilized as a vision backbone, this architecture has yielded state-of-the-art results across various tasks, including semantic segmentation, object detection, and image classification. Swin-Unet, developed by Cao et al. [13], represents the first U-shaped architecture based exclusively on Transformers and tailored for 2D medical image segmentation. Specifically designed for this application, Swin-Unet integrates an encoder, bottleneck, decoder, and skip connection to harness the transformative potential of the transformer architecture to its fullest extent. To enhance fine-grained crack detection, CrackFormer, a Transformer-based network leveraging SegNet’s encoder-decoder architecture alongside innovative attention mechanisms, was introduced by Liu et al. [14]. Cascade CATransUNet, an architecture enhanced with coordinate attention in transformers and featuring a self-cascaded design, was proposed by Chu et al. [15]. To capture the fundamental shapes of fractures in both horizontal and vertical orientations more effectively, CATransUNet, initially devised as a transformer-based architecture for multi-scale feature extraction with integrated coordinate attention, serves as the foundation. Subsequently, a self-cascaded refinement method gradually reconstructs the specific characteristics of identified cracks at both global and local scales. Furthermore, an optimized boundary loss, derived from a combined cascade loss function, is employed to enhance segmentation accuracy at the boundaries.

### 2.3 Mamba-based methods

Recent studies have underscored the significant potential of State Space Models (SSM) in the domain of long sequence modeling, offering innovative solutions to the challenges posed by long-range dependencies in visual tasks. Comparatively, SSMs exhibit superior efficacy in processing extended sequences and in capturing long-range dependencies when juxtaposed with Transformer models. The efficacy of SSMs in visual applications has been corroborated by a plethora of recent research endeavors. Notably, Liu et al. [4] introduced VMamba, a visual state space model characterized by dynamic weighting and expansive global receptive fields. VMamba incorporates a novel Cross-Scan Module, which addresses the discrepancies between one-dimensional array scanning and two-dimensional plane traversal. The Cross-Scan Module enhances the adaptability of SSMs to visual data, ensuring that the breadth of reception remains uncompromised. The Mamba framework has been extensively applied in the field of medical image segmentation, where a multitude of Mamba-based methodologies have demonstrated remarkable efficacy in practical applications. Notably, several Mamba-derived techniques, including U-Mamba [16], VM-Unet [17], Mamba-Unet [18], and SegMamba [19], have significantly augmented the performance of medical image segmentation tasks.

## 3 Methods

### 3.1 State Space Model (SSM)

The State Space Model (SSM) serves as a mathematical framework for delineating the temporal dynamics of evolving systems. Central to the SSM paradigm is the conceptualization of the system’s state at a given instant as a vector within the state space. The progression of this state is meticulously regulated by a system of equations:

h′(t)=Ah(t)+Bx(t) (1)

y(t)=Ch(t)+Dx(t) (2)

Eq (1), the state equation, describes how the system’s state changes over time. Here, h(t) is the system state at time t, h’(t) is the derivative of the state vector h(t) with respect to time, and x(t) is the input signal at time t. Eq (2), the observation equation, describes the relationship between the system’s output and its state. In this equation, y(t) represents the output signal at time t. The matrices A, B, C, and D are the model parameters, known as the state transition matrix, input matrix, observation matrix, and direct transmission matrix, respectively.

### 3.2 Selective State Space Model (S6)

The Selective State Space Model (S6) extends the traditional SSM by incorporating a selection mechanism to enhance the model’s sensitivity and adaptability to input data. The central idea of the S6 model is that not all input data contribute to the current output. Therefore, the state can be selectively updated based on the contextual information of the input data. This selection mechanism allows the model to selectively focus on or ignore certain inputs at different positions in the sequence. It achieves this by treating the model parameters as functions of the input, enabling the model to filter out irrelevant information and retain task-relevant information over the long term.

Within the S6 model, the parameters B, C, and D are treated as functions of the input x(t), thereby enabling the model to adaptively modulate its behavior contingent upon the prevailing input signal:

B=LinearN(x) (3)

C=LinearN(x) (4)

$$S_{\Delta }(x)=BroadcastD(Linear1(x))$$ (5)

Here, LinearN and Linear1 denote parameterized linear transformations, while BroadcastD is an operation used to broadcast information across different dimensions.

To control the selectivity of state updates, the S6 model employs a softplus function to activate or suppress state updates:

$$D=softplus(S_{\Delta }(x))$$ (6)

The softplus function provides a smooth method to control the activation and suppression of states, helping the model maintain stability and selectivity when handling complex sequential data.

### 3.3 2D-Selective-Scan (SS2D) module

To achieve a comprehensive receptive field globally while maintaining computational efficiency, the VMamba model integrates the 2D-Selective-Scan (SS2D) module. This integration is specifically designed to mitigate the inherently non-causal characteristics of visual input data.

**Fig 1:**
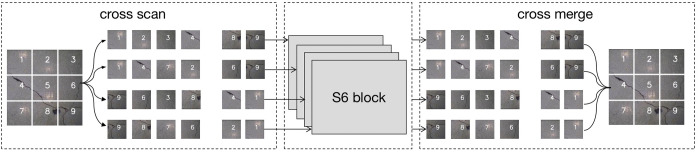
SS2D.

As illustrated in Fig 1, the 2D-Selective-Scan (SS2D) module facilitates the transformation of the image into a one-dimensional (1D) vector by performing a sequential scan across the image in four cardinal directions: top-left to bottom-right, bottom-right to top-left, top-right to bottom-left, and bottom-left to top-right. Subsequently, the resultant set of four 1D vectors is processed independently through S6 blocks. Finally, the four 1D vectors computed by the S6 blocks are fused into a single 2D feature output. This approach, combining the strengths of SSM and the innovative scanning strategy of SS2D, effectively captures spatial dependencies in visual data, achieving a global receptive field while maintaining linear computational complexity.

### 3.4 VSS block

**Fig 2:**
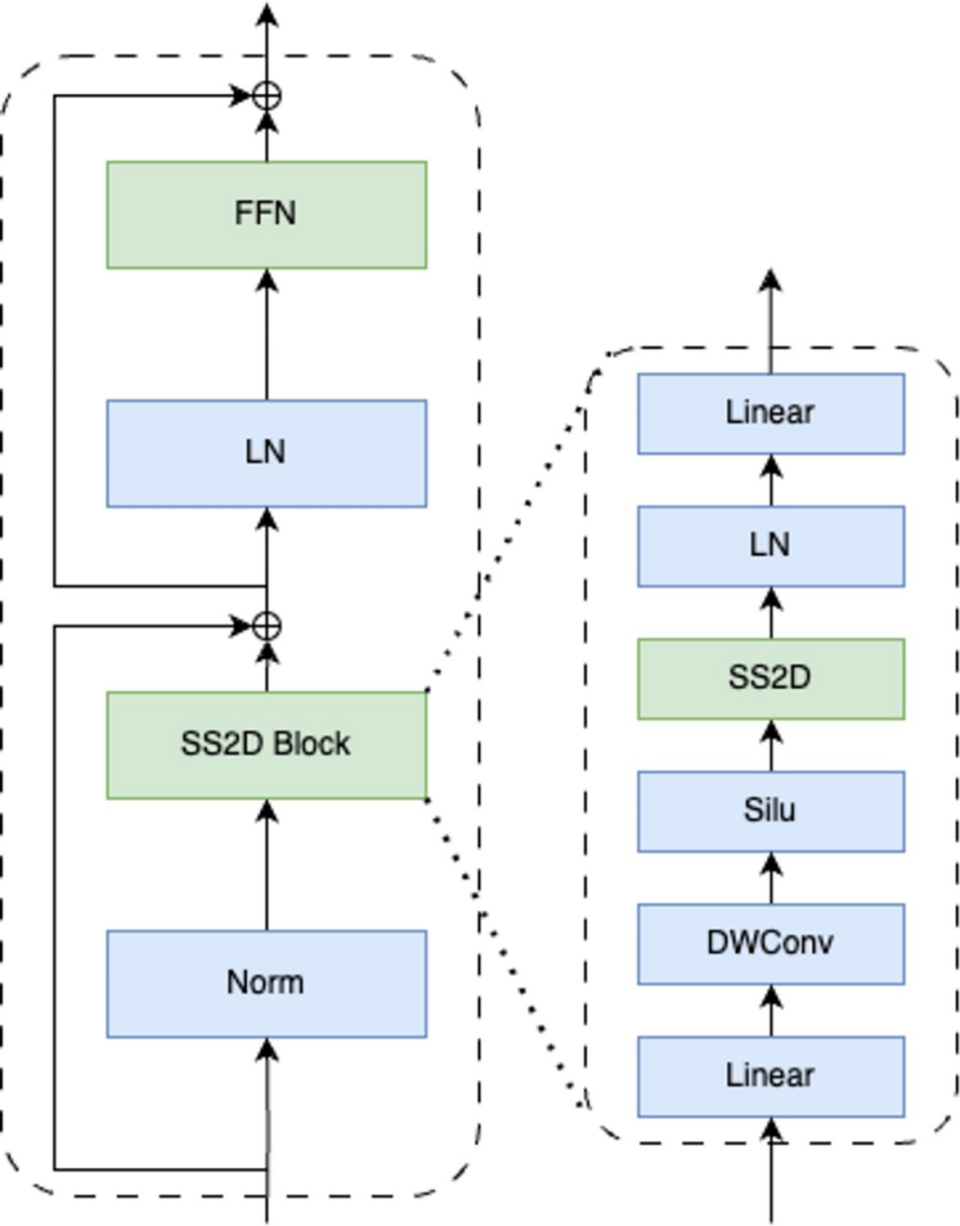
VSS block.

The central component of the VMamba model, as depicted in Fig 2, is the Visual State Space (VSS) block. Initially, layer normalization is applied to split the input into two information streams for the VSS block. In the first stream, the input is processed sequentially through the Silu activation function and a linear layer. In the second stream, the input undergoes processing through a linear layer, a depthwise separable convolution, and an activation function before being transmitted to the 2D-Selective-Scan (SS2D) module for further feature extraction. The features from both streams are then normalized using layer normalization and combined through element-wise multiplication. Subsequently, the characteristics are mixed using a linear layer, and the final output of the VSS block is generated by combining this result with the residual connection.

### 3.5 Mamba-Enhanced HRNet

**Fig 3:**
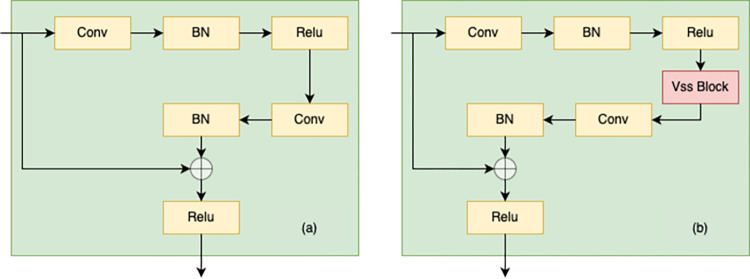
Basic block. (a) Basic block of Origin HRNet. (b) Basic block of ours.

**Fig 4:**
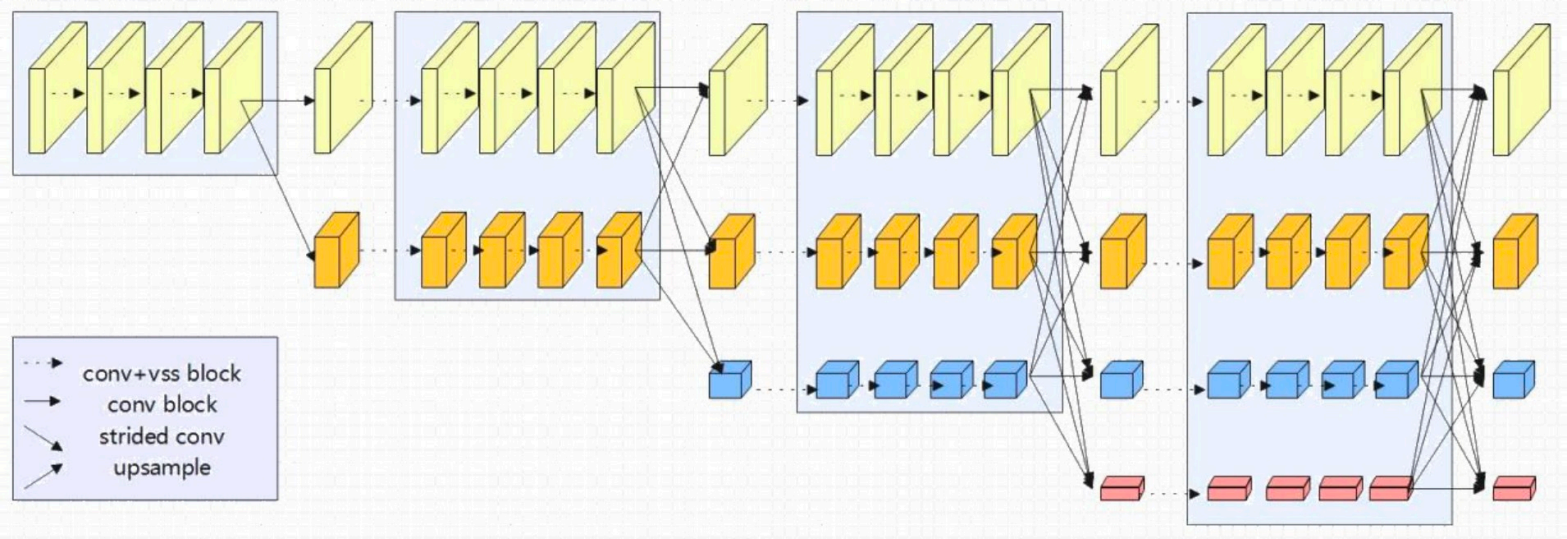
Mamba-Enhanced HRNet.

In this section, a novel network architecture is proposed, termed the Mamba-Enhanced High-Resolution Network (Mamba-Enhanced HRNet). This architecture combines the multi-resolution parallel design of HRNet with the advantages of the Visual State Space (VSS) model from VMamba. The core idea of Mamba-Enhanced HRNet is to replace the residual convolution blocks in HRNet with composite blocks that integrate VSS blocks and convolutional layers, while retaining the multi-resolution parallel structure of HRNet. This design enables features to be learned simultaneously at various resolutions, and the incorporation of VSS blocks improves the network’s capacity to capture global contextual information while preserving computational efficiency. Specifically, the design of Mamba-Enhanced HRNet is as follows:

1. Basic block: In Fig 3, (a) depicts the Basic block devised by HRNet, while (b) showcases the enhanced Basic block proposed in this study. Specifically, we have added VSS blocks to the convolutional residual modules of HRNet. This modification aims to bolster the network’s capacity for detail capture and introduce enhanced global context information acquisition via the VSS blocks.
2. Multi-resolution parallel structure: As depicted in Fig 4, the Mamba-Enhanced HRNet retains the multi-resolution parallel structure design of HRNet, where each stage includes feature maps at different resolutions. This helps in capturing damage features at various scales.

In the decoder part, high-resolution detailed information and low-resolution global information, output from the multi-resolution parallel structure, are effectively combined through skip connections, generating rich multi-scale feature representations. Finally, a 1×1 convolution layer maps the high-resolution feature maps to the number of semantic segmentation categories, producing the final segmentation results.

## 4 Experimental results and analysis

### 4.1 Dataset

To comprehensively evaluate the performance of Mamba-Enhanced HRNet in the semantic segmentation of bridge damage, a carefully constructed dataset encompassing various types of damage images was developed. The dataset integrates the following sources:

- Japan Society of Civil Engineers Bridge Damage Dataset [20]: This dataset includes 5,821 images of bridges, covering multiple types of damage such as corrosion, cracks, free lime, leakage, and spalling.
- Bochum Crack Data Set [21]: This set contains 370 RGB images of concrete cracks, including structures such as bridges, walls, and parking lots, thereby increasing the dataset’s diversity and relevance to practical applications.
- CRACK500 Dataset [22, 23]: Comprising 500 images of concrete road cracks, this dataset provides additional patterns and textures of cracks.

The integration of these datasets aims to create a challenging dataset in terms of diversity, complexity, and multiple perspectives. The model’s robustness is enhanced in complex backgrounds and varying settings, along with its ability to generalize to other forms of damage.

All damage types were uniformly labeled as 1, while background and other non-damage areas were labeled as 0. This simplified binary classification setup is designed to enable the model to focus on distinguishing damage from non-damage areas, thereby improving segmentation accuracy and efficiency. For systematic training and evaluation, the entire dataset was divided into training, validation, and testing sets in a ratio of 8:1:1, respectively. This division was used to train the model, select the best model parameters, and evaluate the model’s generalization ability.

To enhance the model’s generalization ability for bridge damage image recognition, we implemented data augmentation techniques to augment the training dataset. This approach involved the application of random rotations, horizontal and vertical flips, Gaussian blurring, and the random adjustment of brightness and contrast levels to the images. These augmentation strategies not only enriched the diversity of the training samples but also endowed the model with robustness against variations in viewing angles and lighting conditions, which are critical for accurate damage detection.

### 4.2 Evaluation metrics

The performance of the Mamba-Enhanced HRNet in semantic segmentation of bridge damage was quantitatively assessed using the Mean Intersection over Union (Mean IoU) metric. Mean IoU measures the degree of overlap between the predicted and actual regions of damage, with higher values indicating superior segmentation accuracy. The Mean IoU is mathematically defined as follows:

$$MeanIoU=\frac{\sum {IoU}_{i}}{n}$$ (7)

Here, n is the total number of classes, and IoU_i_ denotes the Intersection over Union for the i-th class. The computation for IoU_i_ is given by:

$${IoU}_{i}=\frac{{TP}_{i}}{{TP}_{i}+{FP}_{i}+{FN}_{i}}$$ (8)

In this equation, TP_i_, FP_i_, and FN_i_ respectively denotes the true positives, false positives, and false negatives for the i-th class.

Furthermore, the model’s performance in detecting a variety of damage types was conducted using the recall rate metric.


$${Recall}_{i}=\frac{{TP}_{i}}{{TP}_{i}+{FN}_{i}}$$ (9)

### 4.3 Experimental results and discussion

Mamba-Enhanced HRNet was trained and tested on the constructed dataset, and compared with several other semantic segmentation models, including DeepLabv3+, VM-UNet, HRNet, and HRFormer [24]. During the training phase, the learning rate was initialized at 0.001, and a cosine annealing strategy was employed to gradually reduce the learning rate as training progressed, thereby refining the weight adjustments of the model and enhancing its ultimate performance. Training was conducted using four RTX 4090 GPUs, and considering the balance between memory consumption and training efficiency, a batch size of 16 samples per iteration was set. The following are the experimental results (see Table 1):

**Table 1:** Experimental results on the test set.

	MeanIoU	IoU_1
DeepLabv3+	0.938	0.892
HRNet	0.956	0.924
HRFormer	0.931	0.880
VM-UNet	0.913	0.850
Ours	0.963	0.936

The results indicate that, compared to models such as DeepLabv3+, HRNet, HRFormer, and VM-UNet, Mamba-Enhanced HRNet achieved the highest performance with Mean IoU of 0.963 and IoU_1 of 0.936, demonstrating superior performance in bridge damage detection tasks. Mamba-Enhanced HRNet effectively distinguishes between damaged and undamaged areas, demonstrating the model’s potential for practical applications.

**Fig 5:**
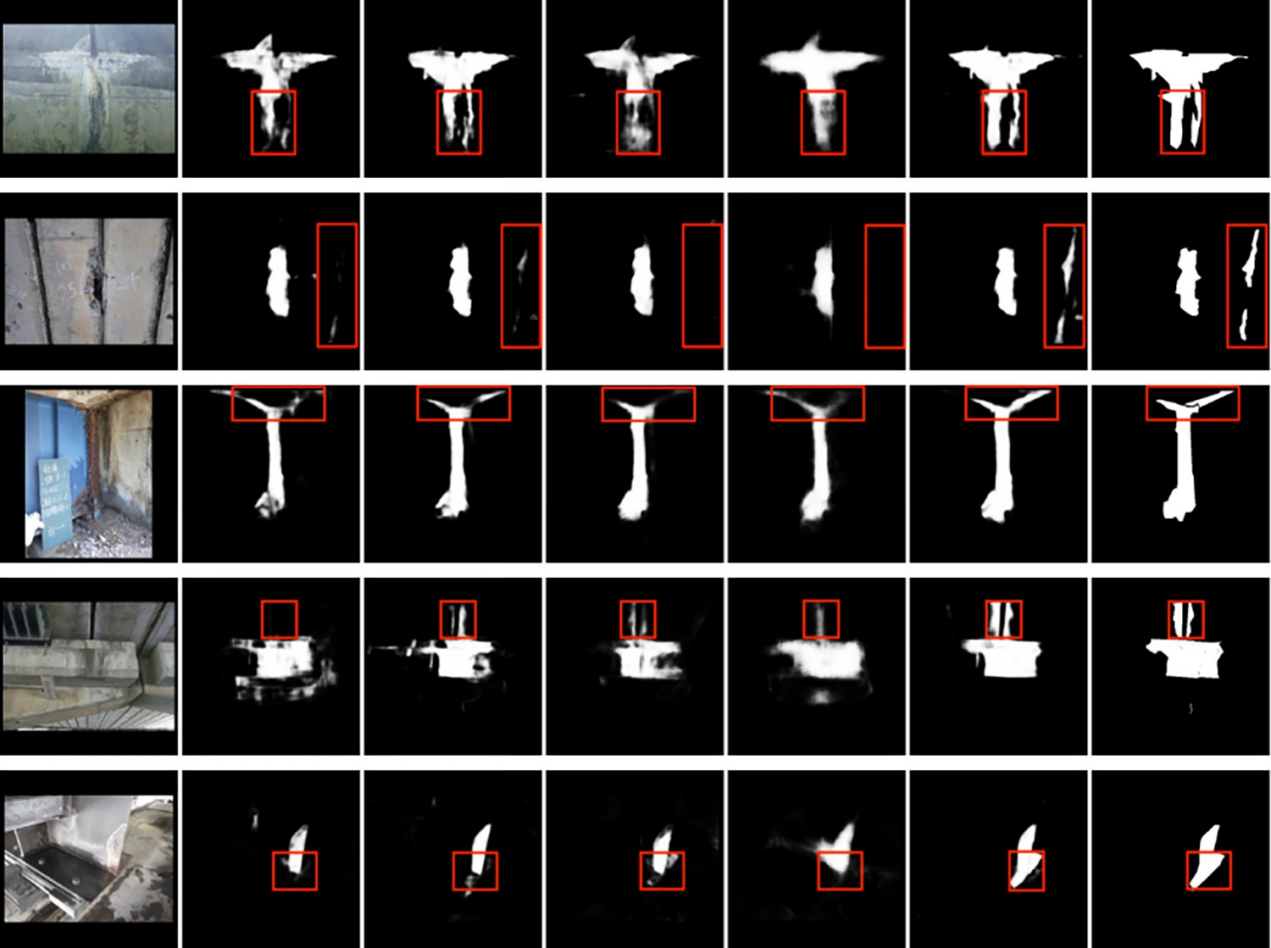
Results of segmentation for each experiment.

Fig 5 illustrates the segmentation results of the five models on the test set, showing original images from left to right, followed by DeepLabv3+, HRNet, HRFormer, VM-UNet, Mamba-Enhanced HRNet, and Ground Truth. Visual inspection of the segmentation results reveals the advantage of Mamba-Enhanced HRNet in capturing details. Even minor cracks and damages are distinctly delineated, which is crucial for bridge maintenance and safety assessment.

In this study, we have constructed a substantial dataset encompassing various types of damage and images from diverse scenarios, categorizing all forms of damage into a single class. To validate the performance of our model in recognizing different types of damage, we assessed it using recall rates on the five damage categories in the Japan Society of Civil Engineers Bridge Damage Dataset (see Table 2).

**Table 2:** Recall rate on various types of damage.

	corrosion	cracks	free lime	leakage	spalling
DeepLabv3+	0.947	0.888	0.919	0.953	0.912
HRNet	0.961	0.909	0.948	0.968	**0.941**
HRFormer	0.912	0.835	0.888	0.924	0.840
VM-UNet	0.923	0.762	0.851	0.883	0.829
Ours	**0.962**	**0.916**	**0.951**	**0.969**	0.938

The experimental results indicate that the Mamba-Enhanced HRNet demonstrated high recall rates for the identification of corrosion, cracks, free lime, and leakage damage categories, while its recall rate for the spalling category was slightly lower than that of the original HRNet model. Overall, the Mamba-Enhanced HRNet exhibited a high degree of accuracy and robustness in the recognition of various damage types.

## 5 Conclusion

The proposed Mamba-Enhanced HRNet provides a novel technical approach for bridge damage detection, achieving significant performance improvement in semantic segmentation tasks for bridge damage detection. Through the multi-resolution parallel design of HRNet and the introduction of VMamba’s VSS module, Mamba-Enhanced HRNet effectively integrates features of different scales and enhances the network’s global receptive field, aiding in capturing long-distance dependencies in images and improving the recognition of small-area damages. Experimental results demonstrate precise segmentation of damage areas and exhibit good generalization capabilities to adapt to various damage types and complex backgrounds.

Despite the outstanding performance of Mamba-Enhanced HRNet in experiments, every model has limitations. For instance, in damage detection under extreme weather conditions, the model may require further adaptation and training. Future work could focus on expanding the dataset to include more diverse environmental conditions and exploring deployment strategies for practical bridge detection.
